# Serum ferritin and admission stroke severity in first-ever acute ischemic stroke: a cross-sectional study

**DOI:** 10.3389/fneur.2025.1683774

**Published:** 2025-11-06

**Authors:** Ying Gao, Xinyi Xu, Haoli Gao, Lei Ren, Yan Wang, Chengboya Zhao, Yongwei Mu, Xiaolu Zhao, Xiaokun Yang, Jihua Liu, Xiudi Lu

**Affiliations:** 1 First Teaching Hospital of Tianjin University of Traditional Chinese Medicine, Tianjin, China; 2 National Clinical Medicine Research Center of Chinese Medicine Acupuncture and Moxibustion, Tianjin, China

**Keywords:** serum ferritin, acute ischemic stroke, NIHSS, neurological deficit, iron metabolism

## Abstract

**Background:**

Iron dysregulation may aggravate ischemic brain injury through oxidative stress and ferroptosis. Serum ferritin (SF) reflects iron storage and inflammation, but its relationship with initial neurological deficit in first-ever acute ischemic stroke (AIS) remains unclear.

**Objective:**

To investigate the association between admission SF levels and stroke severity in patients with first-ever anterior circulation AIS.

**Methods:**

This cross-sectional study included 288 patients with first-ever anterior circulation AIS admitted within 72 h of onset. SF was measured within 24 h of admission. Stroke severity was assessed using the NIHSS; greater neurological deficit was defined as NIHSS > 5. Multivariable logistic regression, sensitivity analyses, and restricted cubic spline (RCS) modeling were performed. Subgroup analyses explored interactions with age, sex, and TOAST subtype.

**Results:**

The median age was 64 years, 66.0% were male, and the median NIHSS score was 3. Patients with NIHSS > 5 had higher SF [231.77 ng/mL (IQR 135.94–303.92)] than those with NIHSS ≤ 5 [175.00 ng/mL (117.12–231.81); *p* = 0.003]. After full adjustment, higher log-SF remained independently associated with NIHSS > 5 (O*R* = 2.12, 95% CI 1.18–3.81; *p* = 0.012). RCS analysis revealed a U-shaped relationship (*P* for non-linearity = 0.029), with stronger associations in patients <65 years (O*R* = 6.17, 95% CI 1.82–20.92; *p* = 0.004) and in small-artery occlusion subtype (O*R* = 4.20, 95% CI 1.41–12.47; *p* = 0.010).

**Conclusion:**

Among patients with first-ever anterior circulation AIS, serum ferritin showed a U-shaped association with neurological deficit. These results warrant validation in larger multicenter studies.

## 1 Introduction

Acute ischemic stroke (AIS) is one of the leading causes of global mortality and long-term disability (1). Cerebral ischemia triggers a cascade of pathophysiological events, including hemodynamic disturbances, energy failure, excitotoxicity, oxidative stress, neuroinflammation, and cell death, which directly contribute to neuronal injury and subsequent neurological deficits in patients (2).

In clinical practice, the National Institutes of Health Stroke Scale (NIHSS) serves as the standardized tool for assessing neurological function. Studies have shown that a higher baseline NIHSS score is positively correlated with more severe brain tissue damage and poorer clinical outcomes (3). However, significant variations in baseline NIHSS scores persist even among patients with similar infarct characteristics (4). This phenomenon reflects the interindividual heterogeneity of AIS and underscores the complexity of pathophysiological responses following ischemic brain injury. It suggests the potential involvement of poorly understood biomolecular networks in the early stages of ischemic damage (5). Therefore, identifying potential factors influencing brain injury may improve our understanding of stroke pathophysiology and facilitate more individualized clinical assessments.

In recent years, the role of systemic iron metabolism in ischemic brain injury has garnered increasing attention (6). Iron is indispensable for neuronal function, oxygen transport, and neurotransmitter synthesis, but its dysregulation can aggravate ischemic damage (7). Under ischemic–hypoxic conditions, cerebral iron homeostasis becomes highly susceptible to disruption. Excessive free iron ions can catalyze the production of reactive oxygen species (ROS) via the Fenton reaction, triggering robust oxidative stress—a key pathophysiological mechanism underlying neuronal death, blood–brain barrier disruption, and neuroinflammatory cascades (8). Notably, ferroptosis, an iron-dependent form of regulated cell death, has been identified as one of the central mechanisms in ischemic brain injury. These findings suggest that iron status may be a critical determinant of early ischemic injury severity.

Serum ferritin (SF), the principal intracellular iron storage protein, is widely measured in routine clinical practice and reflects both body iron stores and acute-phase responses (9). Elevated SF levels have been reported in AIS patients and associated with greater initial NIHSS scores as well as poorer short-term outcomes. For instance, Sakib et al. (10) reported a positive correlation (*r* = 0.71) between SF levels measured within 48 h of admission and NIHSS scores in their cross-sectional study of AIS patients. Similarly, Garg et al. (11) found admission SF levels negatively correlated with the Canadian Neurological Scale (CNS) (*r* = −0.492, *p* < 0.001) and linked to disease prognosis (*p* < 0.001). Sultana et al. (12) also showed positive correlations between admission SF levels and both NIHSS scores and mRS scores at 4 weeks post-stroke (*p* < 0.001). Although this correlation remains controversial (13). This inconsistency may stem from methodological heterogeneity, such as inclusion of both recurrent and first-ever strokes, varying proportions of anterior versus posterior circulation infarctions, and differences in timing of ferritin measurement.

Our study specifically focuses on first-ever anterior circulation AIS patients admitted within 72 h of onset, a more homogeneous population than in many prior reports. This design reduces confounding by prior infarcts and avoids mixing pathophysiological processes of posterior circulation strokes. Moreover, in our hospital setting—a traditional Chinese medicine (TCM) hospital—admitted AIS patients typically present with relatively mild deficits, reflected in our cohort’s median NIHSS score of 3. The predominance of small-artery occlusion (SAO) subtype (49.3%) also reflects the patient profile in this setting, which differs from tertiary neurological centers that often admit larger numbers of severe large-artery or cardioembolic strokes. These features provide both an opportunity and a challenge: the opportunity to clarify ferritin’s role in relatively mild, first-ever strokes, and the challenge of generalizing results to populations with more severe deficits.

## 2 Materials and methods

This retrospective cross-sectional study was approved by the Medical Ethics Committee of the First Affiliated Hospital of Tianjin University of Traditional Chinese Medicine [Approval No.: TYLL2024(K)014]. Informed consent was waived because the study used anonymized medical data.

### 2.1 Participants

We reviewed the medical records of Tianjin University of Traditional Chinese Medicine Affiliated Hospital from Jan to Dec 2024 and identified 288 patients with first-ever AIS. Inclusion criteria:① Age ≥18 years; ② AIS diagnosis confirmed by ICD-10 codes I63.0–I63.9; ③ Underwent magnetic resonance imaging (MRI) within 72 h of symptom onset, showing hyperintense lesions on diffusion-weighted imaging (DWI) and corresponding hypointense signals on apparent diffusion coefficient (ADC) image. Exclusion criteria: ① History of previous cerebral infarction, posterior circulation infarction, or combined anterior–posterior infarction; ② Presence of intracranial hemorrhage, vascular malformation (e.g., cerebral hemangioma), or other non-ischemic cerebrovascular diseases; ③ Active infection (e.g., respiratory, urinary, abdominal, or systemic) or other conditions (e.g., rheumatoid arthritis, lupus, liver failure, dialysis-dependent renal failure, NYHA class IV heart failure) that could confound inflammatory biomarkers; ④ Pre-existing conditions affecting iron metabolism (e.g., iron deficiency anemia, hereditary hemochromatosis, or chronic liver disease); ⑤ Incomplete clinical data or poor-quality MRI images that would hinder assessment of ischemic lesions. Details of the patient screening and exclusion process are presented in Supplementary Figure S1.

### 2.2 Data extraction and variable definitions

Comprehensive data collected within 24 h of admission included. ① Demographics: age, sex, body mass index (BMI), smoking and alcohol history; ② Comorbidities: hypertension, diabetes, atrial fibrillation; ③ Laboratory parameters: Serum ferritin, D-dimer, glucose, hemoglobin, creatinine, and other biomarkers; ④ Inflammatory indices: Systemic immune-inflammation index (SII = platelets × neutrophils/lymphocytes), and Systemic Inflammation Response Index (SIRI = neutrophils × monocytes/lymphocytes) (14); ⑤Imaging: Neuroimaging using MRI was performed to determine lesion size and classify the stroke subtype according to the TOAST criteria, which include Large-artery atherosclerosis (LAA); Cardioembolic (CE); Small-artery occlusion (SAO); Other/undetermined etiology (15).

Stroke severity at admission was assessed using the National Institutes of Health Stroke Scale (NIHSS), which quantifies neurological deficits across 11 domains including consciousness, visual fields, motor strength, and language. Scores were recorded by certified neurologists at the time of hospital admission. In this study, NIHSS values were used to evaluate the initial severity of acute ischemic stroke. Based on clinical practice standards, previous literature (16, 17), and the distribution characteristics of our cohort—where the maximum NIHSS score was 13 and the median was 3—patients with an NIHSS score >5 were categorized as having relatively higher neurological deficits, while those with NIHSS ≤5 were classified as lower deficit cases.

### 2.3 Statistical analysis

Statistical analysis was conducted using R statistical software (version 4.3.3). Missing values were addressed through random forest imputation (Supplementary Table S1), a method chosen for its ability to handle complex relationships between variables. Continuous variables were analyzed using either t-tests or Mann–Whitney U tests, while categorical variables were assessed using Fisher’s exact tests or χ^2^ tests. Because SF showed a skewed distribution, values were log-transformed (logSF) before regression. Multivariate logistic regression was used to evaluate the relationship between SF and stroke severity at admission across three models: Model 1 (unadjusted), Model 2 (adjusted for Age and Sex), and Model 3 (fully adjusted, including Age, Sex, Diabetes, TOAST subtypes, Largest diameter, D-dimer, Hemoglobin, Glucose, Creatinine, and SIRI). Sensitivity analyses were performed to assess the robustness of our findings, including model fitting with the original unimputed dataset and examining nonlinear relationships using restricted cubic splines (4 knots). Subgroup analyses stratified by age, sex, smoking status, alcohol consumption, diabetes, and TOAST subtypes included likelihood ratio tests to evaluate interaction effects. A *p*-value < 0.05 was considered statistically significant.

## 3 Results

### 3.1 Baseline characteristics of the study population

This study ultimately included 288 patients with first-ever AIS. The detailed inclusion and exclusion criteria are given in Supplementary Figure S1. The study population had a median age of 64 years, with males comprising 65.97% (190/288), and a median NIHSS score of 3.00 (1.00–4.00). Based on NIHSS score, all patients were divided into two groups: NIHSS ≤5 (*n* = 238) and NIHSS >5 (*n* = 50). As shown in Table 1, several variables differed significantly between the groups. Patients with NIHSS >5 exhibited a larger lesion diameter [median (Q1, Q3): 1.81 (1.04, 3.06) vs. 1.12 (0.71, 2.09), *Z* = −3.65, *p* < 0.001], a lower proportion of SAO (30.00% vs. 53.36%), and higher proportions of LAA (14.00% vs. 7.56%), CE (10.00% vs. 2.94%), and other cause (46.00% vs. 36.13%). SF levels were significantly elevated in the NIHSS >5 group [231.77 (135.94, 303.92) vs. 175.00 (117.12, 231.81), *Z* = −3.01, *p* = 0.003], while diabetes prevalence was lower (40.00% vs. 60.50%, χ^2^ = 7.09, *p* = 0.008). The NIHSS >5 group also demonstrated higher D-dimer levels [0.51 (0.24, 1.70) vs. 0.29 (0.19, 0.60), *Z* = 3.16, *p* = 0.002], elevated glucose [7.47 (5.65, 11.19) vs. 6.33 (5.40, 8.50), *Z* = 2.27, *p* = 0.023], and reduced creatinine [59.52 (41.61, 75.95) vs. 68.59 (56.33, 82.16), *Z* = 2.66, *p* = 0.008]. Additionally, the systemic inflammation response index (SIRI) was higher in this group [1.56 (0.93, 2.61) vs. 1.09 (0.76, 1.65), *Z* = 2.82, *p* = 0.005]. No significant differences were observed in other variables, including systolic blood pressure, age, fibrinogen, white blood cell count, hemoglobin, platelet count, albumin, total cholesterol, triglycerides, HDL-C, LDL-C, SII, sex, smoking, alcohol consumption, hypertension, or atrial fibrillation (all *p* > 0.05).

**Table 1:** Baseline characteristics and intergroup analysis (NIHSS≤5 vs. NIHSS >5).

Variables	Total (*n* = 288)	NIHSS≤5 (*n* = 238)	NIHSS>5 (*n* = 50)	Statistic	*P*
Age, M (Q₁, Q₃)	64.00 (56.00, 71.25)	65.00 (56.00, 72.00)	63.50 (55.50, 69.50)	*Z* = −1.09	0.276
Sex, *n* (%)				χ^2^ = 0.00	0.996
Female	98 (34.03)	81 (34.03)	17 (34.00)		
Male	190 (65.97)	157 (65.97)	33 (66.00)		
Smoking, *n* (%)				χ^2^ = 0.27	0.606
No	182 (63.19)	152 (63.87)	30 (60.00)		
Yes	106 (36.81)	86 (36.13)	20 (40.00)		
Alcohol consumption, *n* (%)				χ^2^ = 1.72	0.190
No	178 (61.81)	143 (60.08)	35 (70.00)		
Yes	110 (38.19)	95 (39.92)	15 (30.00)		
TOAST, *n* (%)				-	0.004
SAO	142 (49.31)	127 (53.36)	15 (30.00)		
LAA	25 (8.68)	18 (7.56)	7 (14.00)		
CE	12 (4.17)	7 (2.94)	5 (10.00)		
Other cause	109 (37.85)	86 (36.13)	23 (46.00)		
Largest diameter, M (Q₁, Q₃)	1.16 (0.76, 2.32)	1.12 (0.71, 2.09)	1.81 (1.04, 3.06)	*Z* = −3.65	<0.001
NIHSS, M (Q₁, Q₃)	3.00 (1.00, 4.00)	2.00 (1.00, 3.00)	8.00 (7.00, 10.00)	*Z* = −11.23	<0.001
Hypertension, *n* (%)				χ^2^ = 0.12	0.726
No	39 (13.54)	33 (13.87)	6 (12.00)		
Yes	249 (86.46)	205 (86.13)	44 (88.00)		
Diabetes, *n* (%)				χ^2^ = 7.09	0.008
No	164 (56.94)	144 (60.50)	20 (40.00)		
Yes	124 (43.06)	94 (39.50)	30 (60.00)		
Atrial fibrillation, *n* (%)				χ^2^ = 0.55	0.459
No	209 (92.07)	172 (92.97)	37 (88.10)		
Yes	18 (7.93)	13 (7.03)	5 (11.90)		
Systolic blood pressure, Mean ± SD	147.62 ± 20.94	147.00 ± 20.81	150.62 ± 21.48	*t* = −1.11	0.267
Fibrinogen, M (Q₁, Q₃)	2.76 (2.37, 3.28)	2.75 (2.36, 3.25)	2.91 (2.43, 3.44)	*Z* = −1.18	0.240
D-dimer, M (Q₁, Q₃)	0.30 (0.19, 0.73)	0.29 (0.19, 0.60)	0.51 (0.24, 1.70)	*Z* = −3.16	0.002
Ferritin, M (Q₁, Q₃)	180.69 (118.09, 240.43)	175.00 (117.12, 231.81)	231.77 (135.94, 303.92)	*Z* = −3.01	0.003
White blood cell count, M (Q₁, Q₃)	6.81 (5.64, 8.14)	6.76 (5.67, 8.08)	7.49 (5.63, 8.35)	*Z* = −1.10	0.271
Hemoglobin, M (Q₁, Q₃)	156.00 (145.25, 168.00)	156.00 (145.00, 169.00)	156.00 (147.00, 168.00)	*Z* = −0.19	0.851
Platelet, M (Q₁, Q₃)	218.00 (182.25, 256.00)	218.00 (183.00, 253.00)	229.00 (174.00, 268.00)	*Z* = −0.68	0.499
Glucose, M (Q₁, Q₃)	6.39 (5.40, 8.82)	6.33 (5.40, 8.50)	7.47 (5.65, 11.19)	*Z* = −2.27	0.023
Creatinine, M (Q₁, Q₃)	67.73 (54.18, 81.55)	68.59 (56.33, 82.16)	59.52 (41.61, 75.95)	*Z* = −2.66	0.008
Albumin, M (Q₁, Q₃)	40.30 (37.70, 43.20)	40.30 (37.70, 43.00)	40.80 (37.80, 44.80)	*Z* = −1.04	0.300
Total cholesterol, M (Q₁, Q₃)	4.91 (4.05, 5.71)	4.91 (4.07, 5.65)	4.67 (3.76, 5.87)	*Z* = −0.15	0.884
Triglycerides, M (Q₁, Q₃)	1.52 (1.14, 2.50)	1.52 (1.15, 2.40)	1.53 (1.11, 4.35)	*Z* = −0.80	0.425
HDL-C, M (Q₁, Q₃)	1.09 (0.92, 1.34)	1.09 (0.94, 1.31)	1.10 (0.86, 1.58)	*Z* = −0.03	0.977
LDL-C, M (Q₁, Q₃)	2.37 (1.71, 3.25)	2.43 (1.78, 3.33)	2.18 (1.68, 3.13)	*Z* = −0.97	0.332
SII, M (Q₁, Q₃)	521.89 (352.97, 739.21)	515.43 (348.68, 724.04)	599.21 (402.98, 858.06)	*Z* = −1.68	0.093
SIRI, M (Q₁, Q₃)	1.15 (0.78, 1.86)	1.09 (0.76, 1.65)	1.56 (0.93, 2.61)	*Z* = −2.82	0.005

*t*, *t*-test, *Z*, Mann–Whitney test, χ^2^, Chi-square test, −, Fisher exact; SD, standard deviation, M, median, Q₁, 1st quartile, Q₃, 3st quartile.

### 3.2 Multivariate analysis of the association between SF and stroke severity at admission

To evaluate the independent association between SF and stroke severity at admission, we conducted binary logistic regression analyses using an NIHSS score >5 as the dependent variable. The results are presented in Table 2. In the unadjusted model (Model 1), logSF was positively associated with severe stroke (O*R* = 1.99, 95% CI: 1.20–3.31, *p* = 0.008). After adjusting for sex and age in Model 2, the association remained significant (O*R* = 2.12, 95% CI: 1.24–3.62, *p* = 0.006). In the fully adjusted model (Model 3), which accounted for multiple confounders, including age, sex, TOAST classification, and systemic inflammation response index (SIRI), higher logSF values were independently associated with greater NIHSS at admission (O*R* = 2.12, 95% CI: 1.18–3.81, *p* = 0.012).

**Table 2:** Logistic regression analysis of SF and stroke severity at admission.

Variables	*β*	S.E	*Z*	*P*	OR (95%CI)
Model 1					
Log(ferritin)	0.69	0.26	2.67	0.008	1.99 (1.20 ~ 3.31)
Model 2					
Intercept	−4.24	1.64	−2.58	0.010	0.01 (0.00 ~ 0.36)
Log(ferritin)	0.75	0.27	2.75	0.006	2.12 (1.24 ~ 3.62)
Sex					
Female					1.00 (Reference)
Male	−0.38	0.36	−1.06	0.288	0.68 (0.34 ~ 1.38)
Age	−0.02	0.01	−1.17	0.241	0.98 (0.96 ~ 1.01)
Model 3					
Intercept	−1.28	2.73	−0.47	0.640	0.28 (0.00 ~ 58.89)
Log(ferritin)	0.75	0.30	2.52	0.012	2.12 (1.18 ~ 3.81)
Sex					
Female					1.00 (Reference)
Male	−0.06	0.44	−0.15	0.883	0.94 (0.40 ~ 2.22)
TOAST					
SAO					1.00 (Reference)
LAA	0.78	0.62	1.25	0.210	2.18 (0.64 ~ 7.40)
CE	1.99	0.88	2.27	0.023	7.35 (1.32 ~ 41.00)
Other cause	0.38	0.46	0.84	0.403	1.46 (0.60 ~ 3.58)
Diabetes					
No					1.00 (Reference)
Yes	0.44	0.42	1.05	0.293	1.56 (0.68 ~ 3.57)
Age	−0.02	0.02	−1.34	0.179	0.98 (0.95 ~ 1.01)
Largest diameter	0.09	0.14	0.66	0.508	1.10 (0.84 ~ 1.43)
D-dimer	−0.00	0.00	−1.34	0.180	1.00 (0.99 ~ 1.00)
Hemoglobin	−0.02	0.01	−1.37	0.170	0.98 (0.96 ~ 1.01)
Glucose	0.02	0.06	0.40	0.688	1.02 (0.91 ~ 1.15)
Creatinine	−0.02	0.01	−2.62	0.009	0.98 (0.96 ~ 0.99)
SIRI	0.23	0.12	1.99	0.046	1.26 (1.01 ~ 1.58)

OR, odds ratio, CI, confidence interval. Model1: no covariates were adjusted. Model2: Adjust: Sex, Age. Model3: Adjust: Age, Sex, Diabetes, TOAST subtypes, largest diameter, D-dimer, Hemoglobin, Glucose, Creatinine, SIRI.

### 3.3 Sensitivity and subgroup analyses

To assess the robustness of the findings, sensitivity and subgroup analyses were conducted. Initially, the primary regression model was re-estimated using the original dataset without imputation (Table S2). These results confirmed that logSF remained independently associated with greater NIHSS at admission (O*R* = 2.11, 95% CI: 1.12–3.96, *p* = 0.02). The direction of effect was consistent, and the magnitude was comparable to that in the primary analysis (O*R* = 2.12, 95% CI: 1.18–3.81, *p* = 0.012).

**Figure 1:**
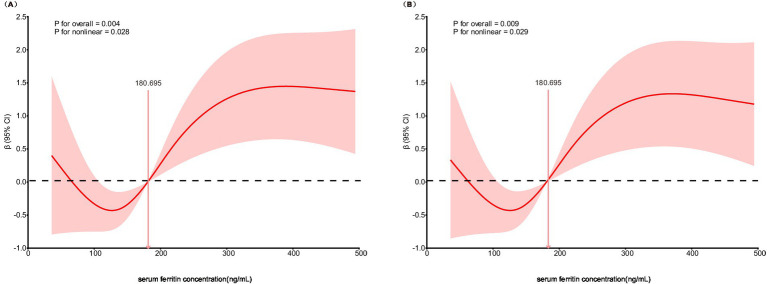
Association of SF concentration and stroke severity at admission in restricted cubic spline model. **(A)** Unadjusted model for SF concentration and stroke severity at admission. **(B)** Multivariable-adjusted model for SF concentration and stroke severity at admission.

The non-linear relationship between SF concentration and admission NIHSS score was evaluated using a restricted cubic spline model embedded within a linear regression framework. This analysis revealed a significant non-linear association (*P* for overall = 0.004; *P* for non-linearity = 0.028). As shown in Figure 1A, the curve demonstrates that stroke severity initially decreases as SF concentration increases up to a certain point, after which severity begins to rise, forming a U-shaped curve. This specific non-linear pattern persisted after multivariable adjustment for confounders (*P* for overall = 0.009; *P* for non-linearity = 0.029), as illustrated in Figure 1B.

To assess the potential modifying effects of key clinical variables, we conducted subgroup analyses using multivariable-adjusted logistic regression models. Table 3 presents the association between SF and greater NIHSS at admission (NIHSS >5) across these subgroups. A statistically significant interaction was found for age (*P* for interactio*n* = 0.007), with a stronger positive association between ferritin and severe stroke in those under 65 years of age (O*R* = 6.17, 95% CI: 1.82–20.92, *p* = 0.004), but no significant association in those aged 65 years or older (O*R* = 1.02, 95% CI: 0.45–2.29, *p* = 0.967). While the association between SF and severe stroke was not statistically significant in females (O*R* = 0.99, 95% CI: 0.35–2.75, *p* = 0.980), it was significant in males (O*R* = 3.38, 95% CI: 1.50–7.65, *p* = 0.003). Among TOAST subtypes, a significant association was observed only in the SAO subgroup (O*R* = 4.20, 95% CI: 1.41–12.47, *p* = 0.010). No significant interactions were found for smoking status, alcohol consumption, or diabetes (all *P* for interaction > 0.05).

**Table 3:** Stratified associations between logSF and stroke severity at admission (multivariable analysis).

Variables	*n* (%)	OR (95%CI)	*P*	*P* for interaction
Age				0.007
<65 years	145 (50.35)	6.17 (1.82 ~ 20.92)	0.004	
≥65 years	143 (49.65)	1.02 (0.45 ~ 2.29)	0.967	
Sex				0.124
Female	98 (34.03)	0.99 (0.35 ~ 2.75)	0.980	
Male	190 (65.97)	3.38 (1.50 ~ 7.65)	0.003	
Smoking				0.647
No	182 (63.19)	2.69 (1.16 ~ 6.24)	0.021	
Yes	106 (36.81)	1.83 (0.68 ~ 4.93)	0.230	
Alcohol consumption				0.457
No	178 (61.81)	2.25 (1.03 ~ 4.92)	0.042	
Yes	110 (38.19)	3.53 (0.88 ~ 14.15)	0.076	
TOAST				0.197
SAO	142 (49.31)	4.20 (1.41 ~ 12.47)	0.010	
LAA	25 (8.68)	0.04 (0.00 ~ 19.81)	0.308	
CE	12 (4.17)	2.36 (0.00 ~ Inf)	1.000	
Other cause	109 (37.85)	1.57 (0.55 ~ 4.47)	0.397	
Diabetes				0.848
No	164 (56.94)	2.20 (0.97 ~ 5.01)	0.059	
Yes	124 (43.06)	1.96 (0.72 ~ 5.31)	0.187	

The multivariable stratified association was adjusted for Age, Sex, Diabetes, TOAST subtypes, largest diameter, D-dimer, Hemoglobin, Glucose, Creatinine, SIRI. Except for the variable that was used for stratification. OR, odds ratio; CI, confidence interval.

## 4 Discussion

In this cross-sectional analysis of first-ever anterior-circulation AIS admitted within 72 h (*n* = 288; median age 64 years; 66.0% male), we found that SF levels within 24 h of admission were independently associated with greater neurological deficit. In the fully adjusted model, log-transformed ferritin was significantly related to higher NIHSS scores (NIHSS > 5) (O*R* = 2.12, 95% CI 1.18–3.81; *p* = 0.012), and the effect persisted in sensitivity analysis (O*R* = 2.11, 95% CI 1.12–3.96; *p* = 0.020). Patients with NIHSS > 5 had higher ferritin values [median 231.77 ng/mL (135.94–303.92)] compared with those with NIHSS ≤ 5 [175.00 ng/mL (117.12–231.81); *p* = 0.003]. These findings are consistent with prior observational studies demonstrating a positive association between admission ferritin and stroke severity measured by NIHSS (18, 19).

It should be noted that the overall cohort represented a relatively mild stroke population, with a median NIHSS score of 3 and a predominance of small-artery occlusion (SAO) subtype (49.3%). This clinical profile likely reflects the characteristics of patients typically admitted to our traditional Chinese medicine hospital, where milder anterior circulation infarctions are more common than severe or posterior circulation strokes. The mild severity and SAO predominance should be taken into account when interpreting the association between ferritin and neurological deficit, as the relationship identified in this study may not fully extrapolate to populations with more severe or large-vessel occlusive strokes. The NIHSS threshold of 5 used to differentiate groups in this study was determined according to the score distribution within our cohort (median 3, maximum 13) and therefore primarily distinguishes patients with relatively higher neurological deficits within a mild-to-moderate spectrum, rather than representing “moderate” stroke severity as defined in large multicenter trials. Future multicenter studies including a broader range of stroke severities and etiologies are needed to validate and generalize these findings.

A non-linear (U-shaped) relationship between SF and NIHSS was identified by restricted cubic spline modeling (overall *p* = 0.004, non-linearity *p* = 0.028), and the pattern persisted after multivariable adjustment (overall *p* = 0.009, non-linearity *p* = 0.029). Stroke severity decreased with rising ferritin up to a moderate level, then increased again at higher concentrations, suggesting that both iron deficiency and iron overload may be detrimental. This bidirectional pattern is biologically plausible, reflecting the “double-edged sword” role of iron in neuronal metabolism. Iron is essential for mitochondrial energy production and neurotransmission, yet excessive catalytic iron amplifies oxidative stress through Fenton chemistry, promotes lipid peroxidation, and triggers ferroptotic neuronal death (7, 20). Conversely, insufficient iron availability can impair oxygen transport and mitochondrial function, reducing neuronal resilience to ischemic stress (21–23). Clinical evidence supports both aspects: iron deficiency after AIS or during stroke rehabilitation has been associated with lower functional capacity and poorer recovery (24). Similarly, anemia, a common correlate of iron deficiency, has been consistently related to worse outcomes after stroke (25). Together, these data lend external coherence to a potential U-shaped association between ferritin and initial neurological severity. Nonetheless, given the exploratory nature of our analysis and the moderate sample size, the non-linear relationship should be regarded as hypothesis-generating and requires validation in larger, prospectively characterized cohorts.

Subgroup analyses indicated significant age interaction (*P* for interactio*n* = 0.007). In patients < 65 years, ferritin was strongly associated with higher NIHSS (O*R* = 6.17, 95% CI 1.82–20.92; *p* = 0.004), whereas no significant association was seen in those ≥ 65 years (O*R* = 1.02, 95% CI 0.45–2.29; *p* = 0.967). Given the median age of 64 in our cohort, this interaction represents a clinically meaningful split. Age-related differences in iron homeostasis and cumulative inflammatory burden (26–28) may explain why ferritin’s effect is clearer in younger patients. In contrast, in older patients, chronic comorbidities and baseline inflammation may overshadow the acute contribution of ferritin.

Sex-stratified analysis showed a significant association in men (O*R* = 3.38, 95% CI 1.50–7.65; *p* = 0.003) but not in women (O*R* = 0.99, 95% CI 0.35–2.75; *p* = 0.980), although the sex interaction was not statistically significant (*P* for interaction = 0.124). In our cohort, with a median age of 64, most women were likely postmenopausal; thus, menstrual iron loss is unlikely to explain the sex difference. Instead, possible explanations include lifelong cumulative iron exposure, baseline ferritin distributions, and sample size imbalance (29). Prior studies similarly show inconsistent sex-specific results, suggesting this requires confirmation in larger datasets (30).

By TOAST classification, the association between SF and NIHSS was significant only in SAO (O*R* = 4.20, 95% CI 1.41–12.47; *p* = 0.010), but not in LAA or CE (P for interactio*n* = 0.197). Interpretation of this finding should consider the overall cohort represented a relatively mild stroke population, with a median NIHSS score of 3 and a predominance of SAO subtype (49.3%). This clinical profile likely reflects the characteristics of patients typically admitted to our institution, where milder anterior circulation infarctions are more common than severe or posterior circulation strokes. This context may partly explain the predominance of SAO and the observed subgroup effect, which should be considered hypothesis-generating rather than definitive. The mild severity and SAO predominance should be taken into account when interpreting the association between ferritin and neurological deficit, as the relationship identified in this study may not fully extrapolate to populations with more severe or large-vessel occlusive strokes. Future multicenter studies including a broader range of stroke severities and etiologies are needed to validate and generalize these findings.

Mechanistically, elevated ferritin indicates higher iron stores. Excess catalytic iron drives hydroxyl radical production via Fenton chemistry, aggravating oxidative stress, blood–brain barrier disruption, and neuronal death (31, 32). Ferroptosis, an iron-dependent regulated cell death pathway, has been increasingly implicated in ischemic injury (33, 34). In addition, ferritin is an acute-phase reactant induced by cytokines such as IL-6, so elevated levels may partly reflect systemic inflammation (35, 36), which itself worsens ischemic brain damage (37, 38). This dual interpretation complicates causal attribution but underscores ferritin’s relevance as a marker of the acute ischemic response. Future studies combining ferritin with inflammatory biomarkers may help distinguish these mechanisms.

### 4.1 Strengths and limitations

Strengths include the restriction to first-ever anterior-circulation AIS with MRI confirmation within 72 h, standardized ferritin measurement within 24 h, exclusion of confounders affecting iron or inflammation, and robust statistical modeling with sensitivity and spline analyses. Limitations include the retrospective, cross-sectional design, which precludes causal inference, and the single-center setting, which may limit generalizability. The lower baseline NIHSS in our cohort (median 3) and high proportion of SAO (49.3%) likely reflect patient selection at a TCM hospital and may influence subgroup findings. Finally, lack of extended iron and inflammatory biomarkers (e.g., transferrin saturation, CRP, IL-6) limits mechanistic interpretation.

## 5 Conclusion

In summary, in patients with first-ever anterior-circulation AIS, higher admission SF was independently associated with greater initial stroke severity, with evidence of a non-linear U-shaped relationship and effect modification by age (<65 years). The predominance of SAO and low median NIHSS in this hospital-based cohort provide important context for interpreting these findings. Further multicenter, prospective studies are needed to validate these results, disentangle the contributions of iron overload and inflammation, and clarify whether ferritin’s effects differ across age, sex, and stroke subtype.

## Data Availability

The original contributions presented in the study are included in the article/Supplementary material, further inquiries can be directed to the corresponding authors.
