# Supplementary material for: Serum ferritin and admission stroke severity in first-ever acute ischemic stroke: a cross-sectional study
Source: Front Neurol. 2025 Nov 6;16:1683774. doi: 10.3389/fneur.2025.1683774 (PMC12631621; doi:10.3389/fneur.2025.1683774)
Supplement: Supplementary file 2 [file Table_2.docx]

### Table S2 Complete Case Analysis of Serum Ferritin and stroke severity at admission: Logistic Regression Analysis

| Variables | β | S.E | Z | *P* | OR (95%CI) |
| --- | --- | --- | --- | --- | --- |
|  |  |  |  |  |  |
| **Model 1** |  |  |  |  |  |
| Log（Ferritin） | 0.69 | 0.26 | 2.67 | 0.008 | 1.99 (1.20 ~ 3.31) |
| **Model 2** |  |  |  |  |  |
| Intercept | -4.24 | 1.64 | -2.58 | 0.010 | 0.01 (0.00 ~ 0.36) |
| Log（ferritin） | 0.75 | 0.27 | 2.75 | 0.006 | 2.12 (1.24 ~ 3.62) |
| Sex |  |  |  |  |  |
| Female |  |  |  |  | 1.00 (Reference) |
| Male | -0.38 | 0.36 | -1.06 | 0.288 | 0.68 (0.34 ~ 1.38) |
| Age | -0.02 | 0.01 | -1.17 | 0.241 | 0.98 (0.96 ~ 1.01) |
| **Model 3** |  |  |  |  |  |
| Intercept | -2.67 | 2.97 | -0.90 | 0.369 | 0.07 (0.00 ~ 23.29) |
| Log（ferritin） | 0.75 | 0.32 | 2.32 | 0.020 | 2.11 (1.12 ~ 3.96) |
| Sex |  |  |  |  |  |
| Female |  |  |  |  | 1.00 (Reference) |
| Male | -0.06 | 0.47 | -0.13 | 0.899 | 0.94 (0.38 ~ 2.35) |
| TOAST |  |  |  |  |  |
| SAO |  |  |  |  | 1.00 (Reference) |
| LAA | 1.10 | 0.66 | 1.67 | 0.094 | 3.00 (0.83 ~ 10.91) |
| CE | 2.13 | 0.91 | 2.35 | 0.019 | 8.46 (1.43 ~ 50.00) |
| other cause | 0.51 | 0.49 | 1.05 | 0.293 | 1.67 (0.64 ~ 4.31) |
| Diabetes |  |  |  |  |  |
| No |  |  |  |  | 1.00 (Reference) |
| Yes | 0.66 | 0.44 | 1.48 | 0.139 | 1.93 (0.81 ~ 4.60) |
| Age | -0.02 | 0.02 | -1.02 | 0.306 | 0.98 (0.95 ~ 1.02) |
| Largestdiameter | 0.12 | 0.14 | 0.83 | 0.408 | 1.12 (0.85 ~ 1.48) |
| Ddimer | -0.01 | 0.00 | -1.32 | 0.185 | 0.99 (0.99 ~ 1.00) |
| Hemoglobin | -0.01 | 0.01 | -0.92 | 0.357 | 0.99 (0.96 ~ 1.01) |
| Glucose | 0.04 | 0.06 | 0.68 | 0.498 | 1.04 (0.92 ~ 1.18) |
| Creatinine | -0.02 | 0.01 | -2.41 | 0.016 | 0.98 (0.96 ~ 0.99) |
| SIRI | 0.18 | 0.12 | 1.44 | 0.150 | 1.20 (0.94 ~ 1.53) |

OR: Odds Ratio, CI: Confidence Interval.
