# Supplementary figures and images for: Serum ferritin and admission stroke severity in first-ever acute ischemic stroke: a cross-sectional study
Source: Front Neurol. 2025 Nov 6;16:1683774. doi: 10.3389/fneur.2025.1683774 (PMC12631621; doi:10.3389/fneur.2025.1683774)

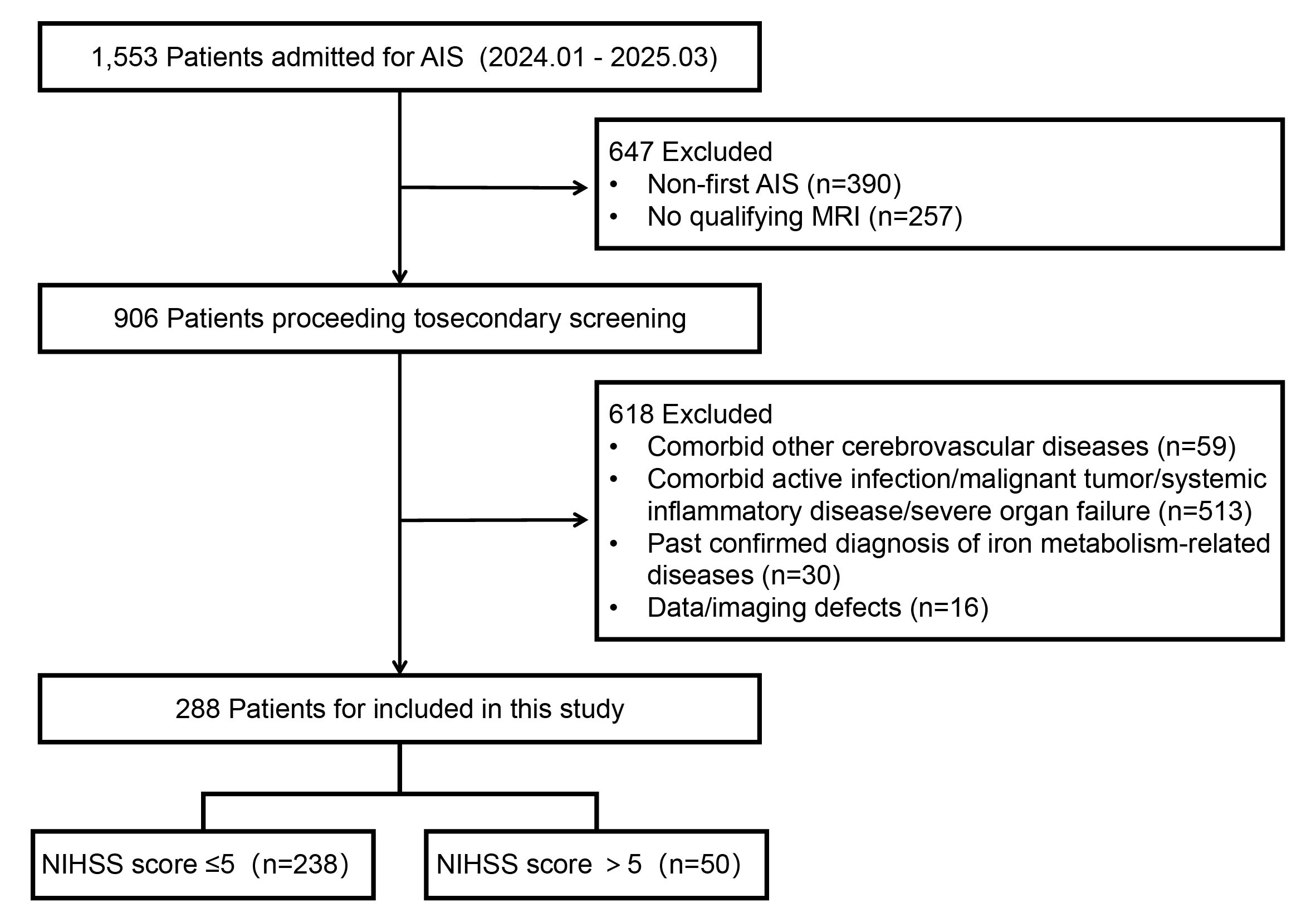

Supplement: Supplementary file 3 [file Image_1.jpeg]
